# Rapid Structural Analysis of a Synthetic Non-canonical Amino Acid by Microcrystal Electron Diffraction

**DOI:** 10.3389/fmolb.2020.609999

**Published:** 2021-01-08

**Authors:** Patrick R. Gleason, Brent L. Nannenga, Jeremy H. Mills

**Affiliations:** ^1^School of Molecular Sciences, Arizona State University, Tempe, AZ, United States; ^2^Center for Molecular Design and Biomimetics, The Biodesign Institute, Arizona State University, Tempe, AZ, United States; ^3^Chemical Engineering, School for Engineering of Matter, Transport and Energy, Arizona State University, Tempe, AZ, United States; ^4^Center for Applied Structural Discovery, The Biodesign Institute, Arizona State University, Tempe, AZ, United States

**Keywords:** microcrystal electron diffraction (MicroED), electron diffraction, transmission electron microscope (TEM), organic synthesis, non-canonical amino acid (ncAA)

## Abstract

Structural characterization of small molecules is a crucial component of organic synthesis. In this work, we applied microcrystal electron diffraction (MicroED) to analyze the structure of the product of an enzymatic reaction that was intended to produce the unnatural amino acid 2,4-dihydroxyphenylalanine (24DHF). Characterization of our isolated product with nuclear magnetic resonance spectroscopy (NMR) and mass spectrometry (MS) suggested that an isomer of 24DHF had been formed. Microcrystals present in the isolated product were then used to determine its structure to 0.62 Å resolution, which confirmed its identity as 2-amino-2-(2,4-dihydroxyphenyl)propanoic acid (24DHPA). Moreover, the MicroED structural model indicated that both enantiomeric forms of 24DHPA were present in the asymmetric unit. Notably, the entire structure determination process including setup, data collection, and refinement was completed in ~1 h. The MicroED data not only bolstered previous results obtained using NMR and MS but also immediately provided information about the stereoisomers present in the product, which is difficult to achieve using NMR and MS alone. Our results therefore demonstrate that MicroED methods can provide useful structural information on timescales that are similar to many commonly used analytical methods and can be added to the existing suite of small molecule structure determination tools in future studies.

## Introduction

The ability to unambiguously characterize the products of chemical reactions is of paramount importance in organic synthesis. A suite of analytical tools including mass spectrometry (MS) and spectroscopic techniques including ultraviolet and visible (UV-vis), infrared (IR) and nuclear magnetic resonance (NMR) are commonly used to characterize organic molecules. These methods are rapid, highly sensitive and—with the exception of MS—non-destructive to the sample. However, because the complete characterization of small molecules often requires the use of many, if not all, of the aforementioned techniques, researchers must possess the necessary expertise to interpret the diverse data generated using each of these analytical methods.

An alternative method that condenses a great deal of information into a single model is to directly determine a small molecule's structure using X-ray crystallography. Molecular structures not only provide the three-dimensional coordinates of each atom in the molecule, but also allow inferences about atom connectivity (i.e., bond number) and molecular packing interactions to be made. Despite these benefits, structural techniques suffer from limitations including the requirement of a large quantity of the small molecule and also that large, well-diffracting crystals are easily formed. These issues are especially problematic for natural products isolated directly from organisms or from very small-scale syntheses; only miniscule quantities of the target compound may be available in both cases. Furthermore, X-ray crystallographic methods are often more time consuming than other analytical methods and are therefore not commonly viewed as high-throughput for rapid small molecule analysis.

In recent years, technological advancements in the field of electron microscopy (EM) have expanded the utility of this technique into the field of protein structure determination (Nogales, 2016; Cheng et al., 2017). A major consequence of this is that access to electron microscopes has increased dramatically both at academic intuitions and through national cryo-EM user facilities. Furthermore, the recent development of techniques such as microcrystal electron diffraction (MicroED) have enabled the determination of the structures of both large biomolecules and small molecules in crystalline form (Gemmi et al., 2019; Nannenga and Gonen, 2019; Nannenga, 2020). Electron diffraction has been successfully applied to a wide variety of samples including proteins, peptides, small organic molecules, and inorganic materials (Mugnaioli et al., 2009, 2012, 2018; Zhang et al., 2013, 2018; Nannenga et al., 2014a,b; Rodriguez et al., 2015; Simancas et al., 2016; van Genderen et al., 2016; Clabbers et al., 2017, 2020; Krotee et al., 2017; Palatinus et al., 2017; Rozhdestvenskaya et al., 2017; Das et al., 2018; Gallagher-Jones et al., 2018; Gruene et al., 2018; Hughes et al., 2018; Jones et al., 2018; Liu and Gonen, 2018; Seidler et al., 2018; Brázda et al., 2019; Dick et al., 2019; Lanza et al., 2019; Warmack et al., 2019; Wennmacher et al., 2019; Xu et al., 2019; Zatsepin et al., 2019; Banihashemi et al., 2020; Levine et al., 2020; Zhu et al., 2020). A major benefit to this technique is that the micro and nanocrystals used for MicroED are several orders of magnitude smaller than those used in conventional X-ray crystallography. Furthermore, in the case of organic molecules, nanocrystals can be even be found in low quantities of synthesized or isolated material and be used directly for the collection of electron diffraction data (Gruene et al., 2018; Jones et al., 2018). Structural analysis using electron microscopes can therefore allow rapid, high-resolution structure determination of organic molecules. Moreover, this technique obviates the need to grow large crystals as is the case for X-ray studies. Thus, MicroED promises to be a powerful tool for organic chemistry and pharmaceutical research (Zhang et al., 2013; van Genderen et al., 2016; Das et al., 2018; Ting et al., 2019; Banihashemi et al., 2020; Levine et al., 2020).

Here we describe the direct characterization of a chemical intermediate obtained during an enzymatic semi-synthesis of a non-canonical amino acid using MicroED. Although evidence from traditional characterization methods (e.g., MS, NMR) provided insight into the structure of the intermediate, these results were incongruous with what would have been expected from an enzyme catalyzed chemical transformation. However, analysis of the intermediate using MicroED methods allowed the high-resolution structure to be determined in ~1 h and confirmed the previous interpretation of the NMR data. Additionally, the MicroED structure indicated that the synthesized product was racemic, which was then confirmed by circular dichroism (CD). This work therefore serves to underscore the power of this burgeoning technique and further suggests that it can reasonably be added to the arsenal of analytical methods that are used to routinely characterize the structures of small molecules in a rapid manner.

## Materials and Methods

### TPL Expression and Purification

A gene encoding tyrosine phenol-lyase (TPL) from *Citrobacter intermedius* was purchased from Integrated DNA Technologies, Inc. (Coralville, IA). The gene was cloned into a pET29b(+) expression vector (Novagen) using Gibson Assembly (New England Biolabs). After Sanger sequence verification, the plasmid was transformed into T7 Express Competent *Escherichia coli* cells (New England Biolabs). Because the pET29b(+) plasmid contains a kanamycin resistance gene, transformed cells were selected on agar plates containing kanamycin (50 μg/mL) to identify clones that contained the expression plasmid.

A single colony of T7 Express cells harboring the expression plasmid was used to inoculate 5 mL of 2xYT media containing kanamycin (50 μg/mL) and supplemented with 1 mM pyridoxal 5'-phosphate (PLP), a required cofactor of TPL. Cultures were incubated for 8 h at 37°C with 250 rpm shaking and ultimately reached an OD_600_ of ~6.0. The 5 mL culture was then used to inoculate an additional 1 L of 2xYT containing kanamycin (50 μg/mL) and PLP (1 mM). This culture was then incubated at 30°C overnight; initial expression tests suggested that the addition of an inducer (e.g., isopropyl β-D-1-thiogalactopyranoside or lactose) was not necessary due to high basal levels of expression from the T7 promoter contained in the pET29b(+) plasmid. Cells were then harvested via centrifugation (5,000 × g, 15 min), resuspended in 50 mL cell lysis buffer (25 mM Tris HCl pH 8.0, 10 mM NaCl, 3 mM β-mercaptoethanol) and stored at −20°C overnight. The frozen cell suspension was thawed at room temperature and incubated at 37°C for 30 min with lysozyme (1 mg/mL). Both MgCl_2_ (20 mM) and DNase (0.2 mg/mL; Sigma Aldrich) were then added to the cell lysate which was subsequently subjected to sonication (20 Hz, 10 min total time, 1 s on, 2 s off). The cell lysate was centrifuged (30,000 rpm for 20 min at 4°C) to remove cell debris.

TPL was then purified on a nickel-nitrilotriacetic acid resin (Ni-NTA, HisTrap FF, GE Healthcare). After loading on Ni-NTA, contaminant proteins were removed by washing the column with five column volumes (CV) of Ni-NTA buffer A (25 mM Tris-HCl pH 8.0, 20 mM Imidazole and 500 mM NaCl) followed by five CV of 90% Ni-NTA buffer A with 10% Ni-NTA buffer B (25 mM Tris-HCl pH 8.0, 500 mM Imidazole and 150 mM NaCl). Proteins were then eluted with five column volumes of 100% Ni-NTA buffer B. Because PLP-bound TPL is bright yellow, fractions that were visibly yellow were collected and concentrated using ultrafiltration (30 kDa molecular weight cutoff, Sartorius Vivaspin). Proteins were then diluted 10-fold with anion exchange (IEC) loading buffer A (25 mM Tris-HCl pH 8.0, 10 mM NaCl) and further purified on IEC resin (HiTrap Q FF, GE Healthcare). The column was washed with five CV of IEC buffer A, followed by five CV of 90% IEC buffer A with 10% IEC buffer B (25 mM Tris-HCl pH 8.0, 500 mM NaCl). Proteins were then eluted with five column volumes of 100% IEC buffer B. Again, protein fractions that were found to be bright yellow were consolidated and concentrated to 500 μl using ultrafiltration as described above. The concentrated protein was then injected onto a size-exclusion column (Superdex 200 increase 10/300 gl, GE Healthcare) and eluted from the column with 25 mM Tris-HCl pH 8.0, 500 mM NaCl. Fractions that were bright yellow were collected, consolidated, and concentrated to a volume of ~1 mL. This protocol yielded ~20 mg of purified protein, which was then refrigerated at 4°C for use in subsequent experimental procedures. The purity of all samples was confirmed via SDS-PAGE (4% stacking/10% resolving, 100 V for 90 min) after each round of purification.

### Small Molecule Synthesis and Purification

In this study, two variations of common protocols for the TPL-based synthesis of the tyrosine analogs were used:

Our first protocol followed the methods described by Kim and Cole (1998) and Kim et al. (2000). To a solution of TPL (190 nM; 30 units) in tris buffered saline (TBS, 25 mM Tris-HCl, pH 8.0, 500 mM NaCl, 5 mM β-mercaptoethanol) was added either resorcinol or phenol (10 mM), sodium pyruvate (60 mM), pyridoxal 5'-phosphate (40 μM), and ammonium chloride (30 mM). The reaction was stirred for 3 days at room temperature. The mixture was acidified to pH 3.0 with acetic acid and filtered over Celite. The filtrate was then extracted with ethyl acetate (3 × 500 mL) to remove excess resorcinol. The entire reaction mixture was then added to prewashed (6 N HCl, water, 6 N NaOH, water) Dowex 50W resin (20 g; Sigma Aldrich), washed with 5 CV of water and eluted with 10% aqueous ammonia. Elution fractions were subjected to a ninhydrin test, which provided a colorimetric indication of the presence of amino acid.

### Synthesis of 2-Amino-2-(2,4-Dihydroxyphenyl)Propanoic Acid

The previously described protocol only indicated the presence of an amino acid in the control (see section Results and Discussion). We therefore employed a second method reported by Seisser et al. (Seisser et al., 2010). In this protocol, purified TPL (190 nM; 30 units) in TBS was added to a dialysis cassette (D-Tube™ Dialyzer Midi, MWCO 6–8 kDa, EMD Millipore) and was placed into 1 L of TBS containing resorcinol (50 mM, Sigma Aldrich) or phenol (50 mM, Oakwood Chemicals), sodium pyruvate (100 mM, Sigma-Aldrich), ammonium chloride (180 mM, Sigma-Aldrich), and PLP (0.04 mM, Alfa Aesar). The reaction was covered with aluminum foil to block ambient light and stirred overnight. A white powder precipitated when either phenol (control) or resorcinol was subjected to the aforementioned conditions; both precipitants tested positive for an amino acid using ninhydrin. The precipitant from the resorcinol reaction was collected on filter paper, washed with water (3 × 50 ml) and dried under vacuum to yield 4.56 g of a white powder, which corresponds to a 46.28% yield. Analysis of the sample was then carried out using ^1^H NMR and ESI-MS: ^1^H NMR (D_2_O, 500 MHz) δ 1.84 (s, 3H), 6.36 (d, 1H, J = 2.10 Hz), 6.41 (dd, 1H, J = 6.41 Hz and 10.80 Hz) and 7.23 (d, 1H, J = 8.60 Hz); ^13^C NMR (D_2_O, 500 MHz) δ 20.51, 59.27, 102.64, 107.41, 114.08, 128.68, 155.27, 158.39, and 174.93; ESI-MS calculated for C_9_H_11_NO_4_ (M): 197.0688 Da; found: 197.0072 Da.

### Thin Layer Chromatography of Products

Thin layer chromatography (TLC) was used as a high-throughput method for the detection of amino acid products. The precipitated reaction products were washed with water, dissolved in 1N NaOH, and run on silica plates (6:3:1 n-butanol:isopropanol:acetic acid). The silica gel plates were then heated to evaporate solvent and ammonia from the reaction mixture. Plates were then dipped in a solution of ninhydrin (1.5% w/v in a 3% v/v acetic acid:n-butanol) and were heated using a hot plate or heat gun; presence of a purple spot after heating was indicative of the presence of an amino acid product.

### MicroED Sample Preparation, Data Collection, and Data Processing

Samples for MicroED analysis were prepared by placing a holey carbon EM grid directly into ~10 mg of the precipitated and dried white powder synthesized as described above. After gentle shaking of the grid in the powder, tweezers were used to extract the grid, and excess material was removed by gently tapping the tweezers. The grid was then loaded into a standard room temperature FEI TEM holder. MicroED data were collected at room temperature on an FEI Tecnai F20 equipped with a TVIPS XF-416 CMOS camera using standard low-dose MicroED data collection procedures (Nannenga et al., 2014a; Shi et al., 2016). Data were collected as the stage rotated at a continuous rate of 0.295 degrees per second and each frame was integrated over 2 s. To process the collected MicroED data sets, they were first converted to SMV format (Hattne et al., 2015). XDS (Kabsch, 2010) was used to index and integrate, and XSCALE was used to merge and scale the data from 3 crystals. The data were phased using SHELXT (Sheldrick, 2015), and the ShelXle (Hubschle et al., 2011) interface was used to refine the structure.

## Results and Discussion

**Figure 1:**
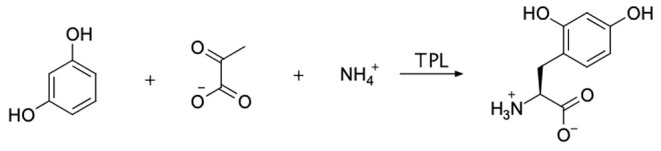
The enzymatic synthesis method employed in this study is shown. Sodium pyruvate, ammonium chloride, and resorcinol were incubated with tyrosine phenol lyase (TPL) with the goal of forming 2,4-dihydroxyphenylalanine (24DHF).

Our primary goal was to synthesize the tyrosine analog, 2,4-dihydroxyphenylalanine (24DHF, Figure 1). A review of the literature suggested a number of possible strategies for achieving this using standard synthetic organic chemistry methods. However, we were drawn to an enzymatic semi-synthesis of this compound using tyrosine phenol lyase (TPL) from *Citrobacter intermedius* because the desired product could be synthesized in a single step from very inexpensive, commercially available starting materials. Namely, in previous reports, TPL was found to catalyze the synthesis of the 24DHF using only resorcinol, pyruvic acid and ammonia (Yamada et al., 1972; Sawada et al., 1975; Nagasawa et al., 1981) as reactants. These data were in contrast another report in which both resorcinol and 24DHF were suggested to represent potent inhibitors of TPL activity (Lambooy, 1954). Nonetheless, given the ease of synthesis, as well as the benefits of enzymatic rather than chemical synthesis (Koeller and Wong, 2001), we chose to explore an enzymatic route to this compound.

We tested two distinct protocols for 24DHF synthesis (see section Materials and Methods) that differed primarily in the concentration of reagents used. In a first protocol (Kim et al., 2000) with low reagent concentrations, no product was observed when resorcinol was used. However, when phenol (the native substrate of TPL) served as a positive control, the expected product, tyrosine, was generated. We then employed a second protocol reported by Seisser et al. (2010) in which high concentrations (50 mM or greater) of the reactants were used. In this case, both the phenol and resorcinol reactions yielded a white precipitate after only 2 h. Notably, both precipitates gave positive results when analyzed with ninhydrin on TLC, which suggested that an amino acid product had formed in both cases.

The product isolated from the reaction containing resorcinol was analyzed by ESI-mass spectrometry (ESI-MS), which indicated that our product had a mass of 197.01 Da. This is consistent with the calculated molecular mass of 24DHF (197.07 Da). Furthermore, analysis of this compound by ^13^C NMR indicated the presence of 9 carbons, which is also consistent with the 24DHF structure (Supplementary Figure 1). However, the ^1^H NMR spectrum (Supplementary Figure 2) was inconsistent with the predicted product. Namely, a singlet that integrated to three protons was observed at 1.84 ppm, which is indicative of a CH_3_ functional group; no methyl groups were expected in the product. Furthermore, neither a CH_2_ (corresponding to protons on the β-carbon) nor a CH proton peak (corresponding to the α-carbon proton) was observed in the ^1^H spectrum. These data suggest that an isomer of 24DHF had been synthesized in lieu of the desired product.

**Figure 2:**
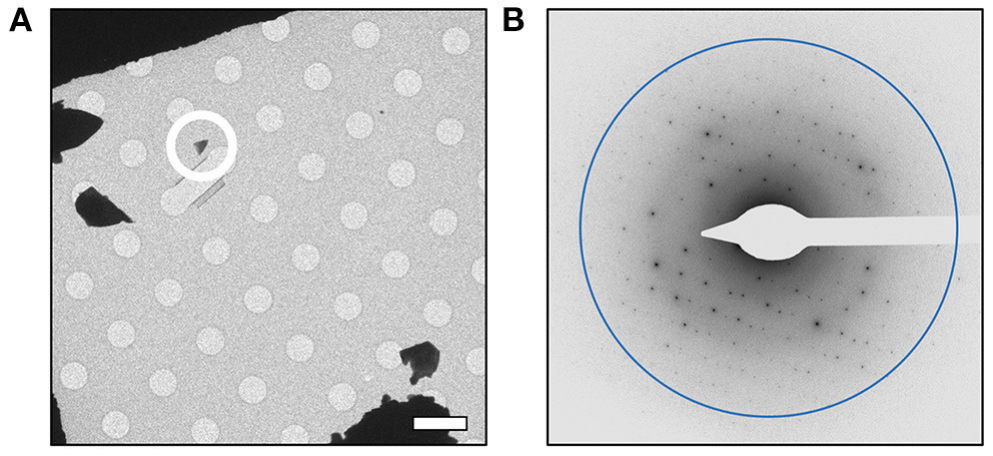
MicroED analysis of 24DPHA. **(A)** Small crystals could be identified on the grid, and a representative crystal used for data collection is circled. All data were collected from crystals of similar size and shape. **(B)** Crystals produced high-resolution diffraction data and continuous rotation data sets were collected from quality crystals. Scale bar in **(A)** represents 5 μm and the resolution ring in **(B)** represents 0.70 Å.

**Figure 3:**
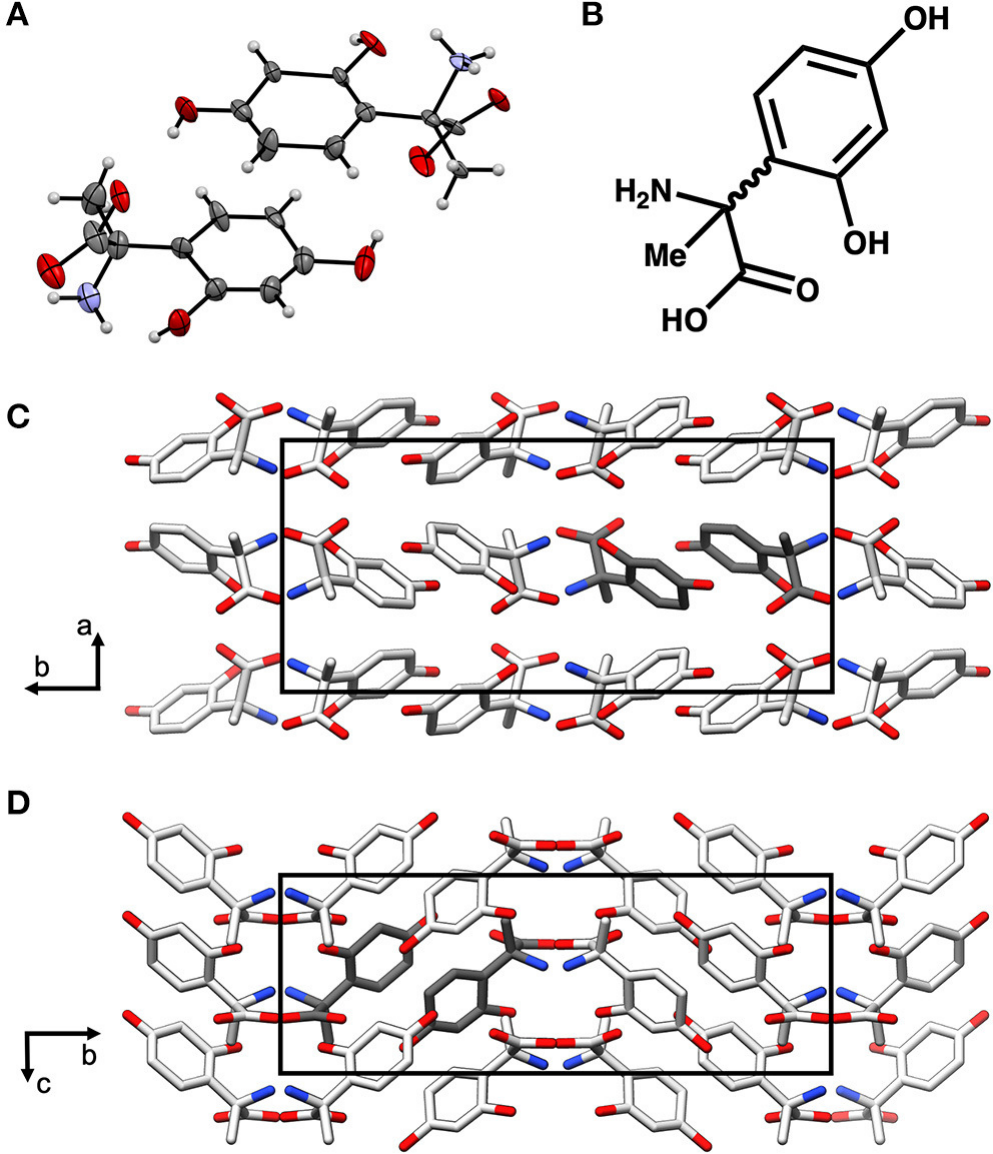
The MicroED structure of 24DHPA **(A,B)**. The space group of the crystal is Pca2_1_ and contains two enantiomers in the asymmetric unit (dark gray), and the packing of the crystals shows hydrogen bonding between adjacent molecules **(C,D)**. The unit cell is shown as a black outline in **(C,D)**.

In order to further characterize the isolated compound, we used MicroED for high-resolution structure determination. There are several methods for preparing small molecule samples for the TEM, including applying powdered sample directly to the grid, applying a crystalline suspension to the grid followed by drying, and direct crystal growth on the grid by solvent evaporation (van Genderen et al., 2016; Gruene et al., 2018; Jones et al., 2018; Banihashemi et al., 2020; Levine et al., 2020). For this analysis, preparing the EM grids with the sample was performed by simply placing the grids in a small amount of material as described above, a process which took less than 1 min. Upon loading the sample into the TEM, extremely small crystals could be found on the grid (Figure 2A), and the many of these diffracted to high-resolution (Figure 2B). Diffraction data were quickly collected from several crystals that all showed similar size and morphology. Ultimately, using standard procedures in XDS (Kabsch, 2010), data from 3 of the highest quality MicroED data sets were processed, all in space group 16 with similar unit cell parameters (Supplementary Table 1). These data were then merged into a final data set that was 62.1% complete (Table 1). Despite the low completeness, a solution could be found using SHELXT (Sheldrick, 2015), which allowed us to determine and refine the structure of the compound (Figures 3A,B). The structural model shows that instead of 24DHF, the resorcinol was added to the α-carbon to yield 2-amino-2-(2,4-dihydroxyphenyl)propanoic acid (24DHPA) (Figure 3B), which confirmed the unexpected findings from NMR.

**Table 1:** Data collection and refinement statistics.

**Data collection**	
Excitation voltage	200 kV
Wavelength (Å)	0.0251
Number of crystals	3
**Data processing**	
Space group	Pca2_1_
Unit cell length a, b, c (Å)	10.40, 22.80, 8.16
Angles α = β = ɤ (°)	90
Resolution (Å)	0.62
Number of reflections	24,851
Unique reflections	2,990
R_obs_ (%)	22.1 (96.0)
R_meas_ (%)	23.5 (114.9)
I/σ_*I*_	5.32 (1.00)
CC_1/2_ (%)	97.8 (51.3)
Completeness (%)	62.1 (42.3)
**Structure refinement**	
R1	0.1839 (0.1549 for F_o_ > 4σ)
wR2	0.4394
GooF	1.288

**Figure 4:**
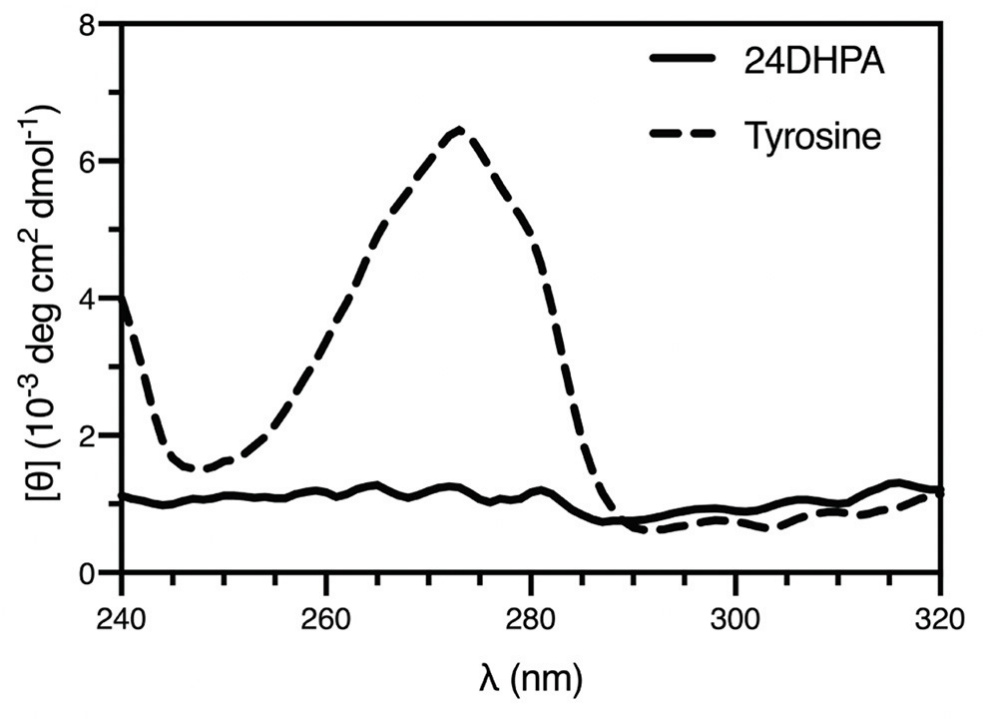
CD spectra of the racemic mixture of 2-amino-2-(2,4-dihydroxyphenyl)propanoic acid (24DHPA) and L-tyrosine (10 mM in 1 mM cuvette).

An additional finding from the high-resolution structure was that both enantiomers were observed in the asymmetric unit, suggesting that the compound was present as a racemic mixture. This was subsequently confirmed using circular dichroism (Shinitzky et al., 2004) (CD, 10 mM sample in 300 μL 1 M HCl), which resulted in a featureless spectrum (Figure 4). Because racemic mixtures of compounds are difficult to identify with NMR without first subjecting the sample to a chiral derivatization agent, the use of electron diffraction has the advantage of providing this information concurrently with the determination of the structure.

Furthermore, the fact that a racemic mixture of products was observed seems inconsistent with a TPL-based enzymatic synthesis. Namely, because TPL's native substrate is the L-amino acid tyrosine, a single product with L- stereochemistry should have been produced using these conditions. As mentioned above, both resorcinol and the desired 24DHF product have been previously suggested to be inhibitors of TPL (Lambooy, 1954). That we observed a racemic product supports the possibility that either the resorcinol or a small amount of 24DHF synthesized by TPL in an initial reaction may have inhibited TPL and precluded enzymatic synthesis by blocking access to the active site. Although beyond the scope of this study, the mechanism of the reaction that led to the aberrant product could warrant additional inquiry.

## Conclusion

In this work, we demonstrate the utility of MicroED as a rapid method for determining the structure of a synthetic product. Although standard analytical techniques (e.g., ^1^H NMR) indicated that our isolated product was not 24DHF as we had set out to make, the MicroED data rapidly confirmed our interpretation. Furthermore, the structure of the aberrant product that was determined using MicroED methods immediately suggested the presence of a racemic mixture of products, which obviated the need to carry out an analysis of the stereochemical properties of the product using chiral separation or polarimetry. The MicroED process, including sample preparation, data collection, data processing and structure refinement, was completed in ~1 h and yielded a structure at 0.62Å resolution. Thus, our results further confirm that MicroED represents a rapid method of gaining insight into the structural features of organic molecules that may prove difficult to obtain using more traditional methods. While it is unlikely that structure determination by electron diffraction will supplant more commonly used analytical techniques, we believe it could be reasonably added to the existing set of analytical tools in future studies.

## Data Availability Statement

The raw data supporting the conclusions of this article will be made available by the authors, without undue reservation.

## Author Contributions

All authors contributed to the planning and conducting of the experiments, the analysis of results, and writing the manuscript.

## Conflict of Interest

The authors declare that the research was conducted in the absence of any commercial or financial relationships that could be construed as a potential conflict of interest.

## Supplementary Material

Supplementary Figure 1 ^13^C NMR spectrum of 2-amino-2-(2,4-dihydroxyphenyl)propanoic acid (24DHPA) in D_2_O.

Supplementary Figure 2 ^1^H NMR spectrum of 2-amino-2-(2,4-dihydroxyphenyl)propanoic acid (24DHPA) in D_2_O.

Supplementary Table 1 Data processing statistics for individual crystals using XDS and XSCALE.
