# Supplementary figures and images for: Rapid Structural Analysis of a Synthetic Non-canonical Amino Acid by Microcrystal Electron Diffraction
Source: Front Mol Biosci. 2021 Jan 8;7:609999. doi: 10.3389/fmolb.2020.609999 (PMC7821094; doi:10.3389/fmolb.2020.609999)

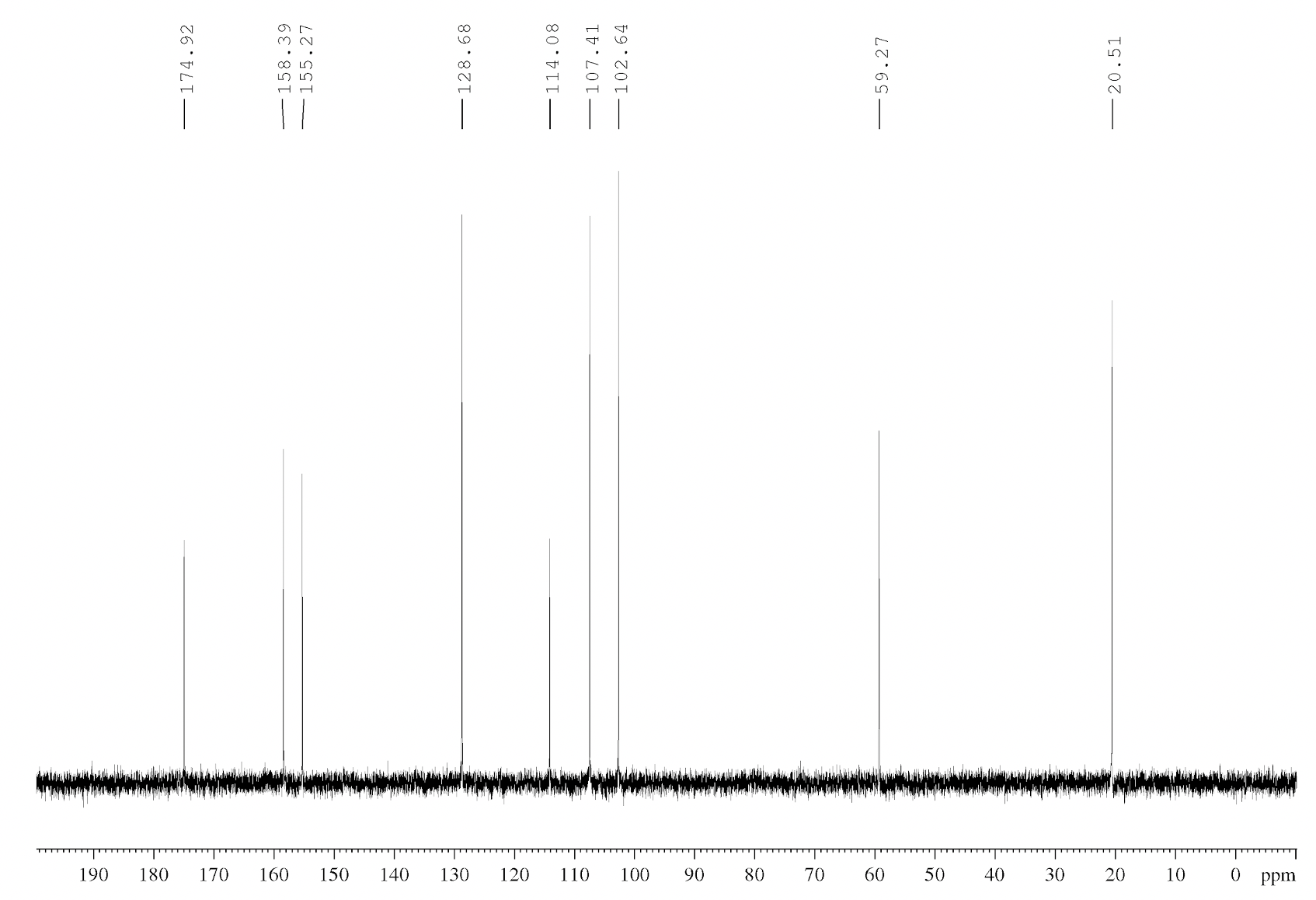

Supplement: Supplementary Figure 1 — 13C NMR spectrum of 2-amino-2-(2,4-dihydroxyphenyl)propanoic acid (24DHPA) in D2O. [file Image_1.TIF]

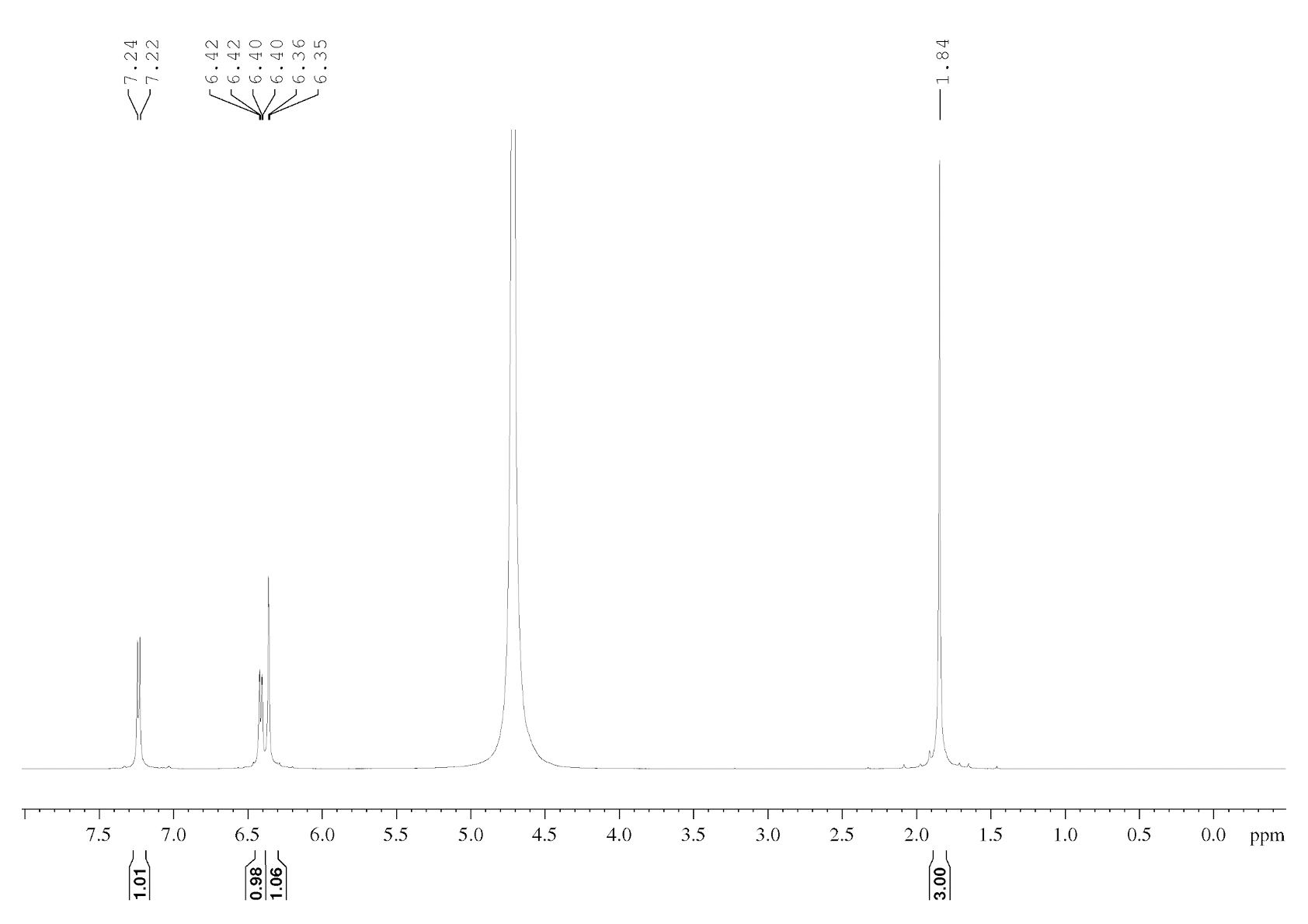

Supplement: Supplementary Figure 2 — 1H NMR spectrum of 2-amino-2-(2,4-dihydroxyphenyl)propanoic acid (24DHPA) in D2O. [file Image_2.TIF]
