# Supplementary material for: Rapid Structural Analysis of a Synthetic Non-canonical Amino Acid by Microcrystal Electron Diffraction
Source: Front Mol Biosci. 2021 Jan 8;7:609999. doi: 10.3389/fmolb.2020.609999 (PMC7821094; doi:10.3389/fmolb.2020.609999)
Supplement: Supplementary Table 1 — Data processing statistics for individual crystals using XDS and XSCALE. [file Table_1.docx]

**Table S1.** Data processing statistics for individual crystals using XDS and XSCALE

|  | Crystal 1 | Crystal 2 | Crystal 3 | Merged Data Set |
| --- | --- | --- | --- | --- |
| Space group | P222 | P222 | P222 | P222 |
| Unit cell length a, b, c (Å) | 8.16, 10.40, 22.80 | 8.12, 10.43, 21.92 | 8.25, 10.49, 22.31 | 8.16, 10.40, 22.80 |
| Angles α = β = ɣ (°) | 90 | 90 | 90 | 90 |
| Resolution (Å) | 0.66 | 0.62 | 0.78 | 0.62 |
| Number of reflections | 10,825 | 8,066 | 6,038 | 24,851 |
| Unique reflections | 2,279 | 2,764 | 1,368 | 2,990 |
| R_obs_ (%) | 16.3 (78.9) | 8.6 (106.2) | 14.7 (102.2) | 22.1 (96.0) |
| R_meas_ (%) | 18.4 (89.1) | 8.1 (97.7) | 16.9 (116.8) | 23.5 (114.9) |
| I/σ_Ι_ | 4.13 (1.19) | 5.98 (1.07) | 4.43 (1.28) | 5.32 (1.00) |
| CC_1/2_ (%) | 98.6 (45.7) | 99.7 (51.2) | 99.1 (65.8) | 97.8 (51.3) |
| Completeness (%) | 55.8 (39.9) | 59.6 (56.0) | 54.9 (54.3) | 62.1 (42.3) |
